# Vertical integrated service model: an educational intervention for chronic disease management and its effects in rural China – a study protocol

**DOI:** 10.1186/s12913-018-3355-8

**Published:** 2018-07-20

**Authors:** Shaofan Chen, Bo Burström, Vibeke Sparring, Dongfu Qian

**Affiliations:** 1 Karolinska Institutet, Department of Learning, Informatics, Management and Ethics, Stockholm Centre for Healthcare Ethics, Health Outcomes and Economic Evaluation Research Group, SE-17177 Stockholm, Sweden; 2 Karolinska Institutet, Equity and Health Policy Research Group, Department of Public Health Services, SE-17177 Stockholm, Sweden; 3 Stockholm County Council, Centre for Epidemiology and Community Medicine, P.O. Box 45436, SE-10431 Stockholm, Sweden; 4 Karolinska Institutet, Medical Management Centre, Department of Learning, Informatics, Management and Ethics, SE-17177 Stockholm, Sweden; 5 School of Health Policy and Management, Nanjing Medical University, No. 101 Longmian Avenue, Nanjing, 211166 China; 6 Creative Health Policy Research Group, Nanjing Medical University, No. 101 Longmian Avenue, Nanjing, 211166 China; 7 Center for Health Policy Studies, Nanjing Medical University, No. 101 Longmian Avenue, Nanjing, 211166 China

**Keywords:** China, Rural, Vertical integration, Chronic disease, Healthcare reform

## Abstract

**Background:**

Chronic diseases are becoming a huge threat to the Chinese health system. Although the New Round of Medical Reform aims to improve this, the chronic disease management in rural China is still worrying as it relies highly on hospital care instead of primary care. The vertical integrated care model has proven to be effective for chronic disease patients in many high-income countries, while few studies have been conducted in China. In this project, vertical integrated care will be applied to optimize the care of patients with type 2 diabetes mellitus (T2DM) and primary hypertension in rural China, and to shift the care from hospital to primary care.

**Methods:**

An educational intervention was conducted in three pilot counties in Jiangsu province, a high-income province in southeast China. The intervention was based on the model of vertical integrated care between the three-levels of healthcare institutions. In the pilot counties, 22 townships were included (11 in the intervention and control groups, respectively). Service teams assembled by the local health bureaus implemented the intervention which provides services for both patients and healthcare professionals. Questionnaire interviews (*n* = 4259) and medical records were used to collect patient data (physiological measures, health-related quality of life, satisfaction with care). Data from healthcare professionals (*n* = 282) was gathered through questionnaires and in-depth interviews (knowledge about chronic diseases, general procedure of diagnosing and registering, chronic disease management situation, perceptions of chronic disease treatment and prevention). Baseline data were collected before the start of the intervention in Nov 2015, follow-up data in Oct-Nov 2016, and final data completed in Jul-Aug 2017.

**Discussion:**

The intervention has been conducted smoothly and gotten support from patients, healthcare institutions and local health authorities. The research team anticipates that the vertical integrated model will improve patients’ health, satisfaction with care, and their understanding of their chronic disease. We also anticipate that healthcare professionals can acquire more information about chronic diseases and improve their strategy for providing good quality care for patients.

**Trial registration:**

ISRCTN13319989 Registration date: 4th April, 2017.

**Electronic supplementary material:**

The online version of this article (10.1186/s12913-018-3355-8) contains supplementary material, which is available to authorized users.

## Background

A great challenge for healthcare systems is the rapid population aging and increasing morbidity of chronic disease all over the world [1–3]. This is also the case in China where 19% of the population in 2015 suffered from different kinds of chronic diseases [4]. The situation in rural areas is even worse, for example, the prevalence rate of diabetes in rural China increased 6.6% from 2005 to 2013, which is much faster than the prevalence rate in urban areas [5]. According to a current study, although diabetes was more common in urban areas, it was associated with greater excess mortality in rural areas [6].The previous strategy for chronic disease management in China has focused much more on the costly hospital care, instead of primary care and self-management in rural areas [7–9]. The New Rural Cooperative Medical Service (NRCMS) is the main health insurance type for rural residents. In accordance with the provisions,and within the limits of expenses that can be reimbursed, the reimbursement does not cover the current treatment fee for patients with diabetes and hypertension [10, 11]. For example, although the reimbursement rate can reach 60% for visiting village clinics for outpatients in rural areas of Jiangsu Province, the prescription medicine reimbursement may not exceed 10 CNY for each visit [12]. Access and utilization of health services have improved, but catastrophic health expenses for patients still prevail [13]. This may have an impact on patients’ economic burden in relation to their health and also on their healthcare seeking behaviour [14, 15].

In 2008, a steering group for healthcare reform was established inside the State Council of China [16]. One year later, a new round of healthcare reform was officially launched after the Central Government published a guidance book, which emphasized the importance of a healthcare system and health services both for urban and rural areas [17]. In 2012, the former Ministry of Health (National Health and Family Planning Commission, NHFPC) issued the “*The Plan for Chronic Disease Prevention in China (2012-2015)*”, which has become the guiding principle of prevention and treatment for chronic disease in China [18]. After 2015, another guideline has been published: “*Long Term Planning for Chronic Disease Prevention in China (2017 - 2025)*”, which demonstrated the determination of improving chronic disease management in China [19]. Moreover, “*Basic Public Health Services*” has been announced both by former Ministry of Health and Ministry of Finance [20]. Among all 12 projects, the management service for patients with diabetes, hypertension, and mental disease have been included. The basic services for the chronic diseases mentioned in the documents (such as regular follow-up, and transfer treatment, etc.) are free of charge [20].

The current chronic disease management in China still relies on hospital care, while primary care works as supplementary measures for patients [21]. The most common support of medical service for patients with chronic conditions comes from hospitals. Primary healthcare institutions (community health centre, township health centre, and village clinic, etc.), however, can only provide very basic care, such as blood pressure or blood glucose measuring. One of the most important causes for the hospital-centred management is the lower level of professional ability and equipment in those primary care institutions [21]. The over dependence on hospital care leads to low effectiveness of primary care, and results in long waiting times and heavy financial burden for patients as well [7]. Community care - which is a different phrase but the same concept as primary healthcare - plays a vital role in chronic disease management, is still insufficient in China [22]. A study focusing on the community health management service for elderly patients with chronic conditions in a south-eastern China city showed that the service based on community care had poor availability for patients [23]. Meanwhile, the regulation of household registration policy in China prevented patients from getting access to the local healthcare system where they actually lived [23]. Apart from the obstacles mentioned above, some other issues and problems remain to be solved. For example, the immaturity of management network, lack of political, legal, and financial support, too much focus on treatment instead of prevention, and poor communication between institutions for chronic disease management [21].

The integrated care model is becoming increasingly popular and may be a possible way of improving chronic disease management. It is defined as a process which is based on the model that different types and levels of institutions offer integrated medical and social service for patients [24]. It also links and coordinates care delivered by different healthcare levels, of primary and secondary care [24]. Integrated care can be divided into three types: macro-level integration between systems, which is also known as horizontal integrated care; meso-level integration between healthcare institutions, or so-called vertical integrated care; and micro-level integration in clinic [25]. This study focuses on vertical integrated care and collaboration in chronic disease services between three-level healthcare institutions in rural China. The research team adopted the collaborative care model (CCM) – which is one type of vertical integrated care – to design the intervention [7]. CCM concentrates on providing multiple components of services for patients based on communication and cooperation between members in a service team [7]. In this model, a multidisciplinary group of healthcare delivery professionals provides care in a coordinated fashion and are empowered to work at the top of their professional training [26]. The current CCM projects shows a number of advantages, such as clear division of work and responsibility, rapid response to patients’ demands, better cooperation between different levels of the healthcare system, and improvement of service delivery system [7].

Many high-income countries apply CCM in healthcare service for chronic disease patients, especially in primary care for mental disease patients [7]. Evidence shows that collaborative care is more effective than usual care for treating chronic conditions [26, 27]. CCM was first adapted in primary care in the United States, and evidence has shown that CCM is effective for improving primary care quality for patients with depression within Veterans Affairs (VA) [7, 26]. Studies on CCM in the UK also show improvement of service delivery and service quality for co-morbid depression and physical health problems [28]. Integrated care and CCM are both new concepts in healthcare in China, but preliminary studies have been conducted [27, 29]. A systematic review of research on chronic disease management based on CCM was published in 2014, and most of the studies included in this review focused on diabetes and hypertension care [7]. The 33 articles reviewed concentrated on implementing CCM into primary healthcare for patients with chronic conditions in different settings in China. Only one study was conducted in a rural area, indicating the lack of efforts for chronic disease patients who live outside urban areas. Meanwhile, the review also concluded that the current design of CCM is still in its infancy in China. For instance, 33% of the included studies clearly described the components and work division for members in service team, and only 27% illustrated the specific communication strategies (such as regular meetings, seminars, working chat group on instant messaging software, etc.) of the service team. Some studies also reported implementation of other models similar to CCM. For example, a regional medical collaboration has been implemented in Changning District, Shanghai [30, 31]. In Xicheng District of Beijing, a “health services community” - a collaboration which contains primary care institutions and higher level hospitals, and aims to improve the primary care with the support of higher level hospitals - has been established for diabetes patients [32]. Both of these implementations have shown positive results in improving transfer treatment and reducing patients’ out of pocket expenditure [31, 32].

In order to integrate services for chronic disease, many urban areas in south-eastern China have adopted the idea of a “Hospital-Community” model with the aim to deepen the cooperation between hospital care and primary care [32, 33]. However, studies of vertical integrated care models and CCM for chronic disease management in rural China are still lacking [34], which indicates a great need for conducting studies in rural areas, including chronic disease management.

## Methods/design

### Research aim

This project aims to optimize the care of patients with type 2 diabetes mellitus (T2DM) and primary hypertension in rural areas through an educational intervention directed to healthcare professionals, to shift the care of patients with T2DM and primary hypertension from hospital to primary care services, and to improve vertical integration in healthcare.

### Specific research objectives:

- To evaluate the effectiveness of implementing a vertical integrated model in rural China, from the perspective of patients and health professionals.
- To implement an integrated motivation model for medical professionals, and to evaluate its influence.
- To evaluate the intervention in rural China and to build a primary care management model for patients with chronic disease.

**Fig. 1:**
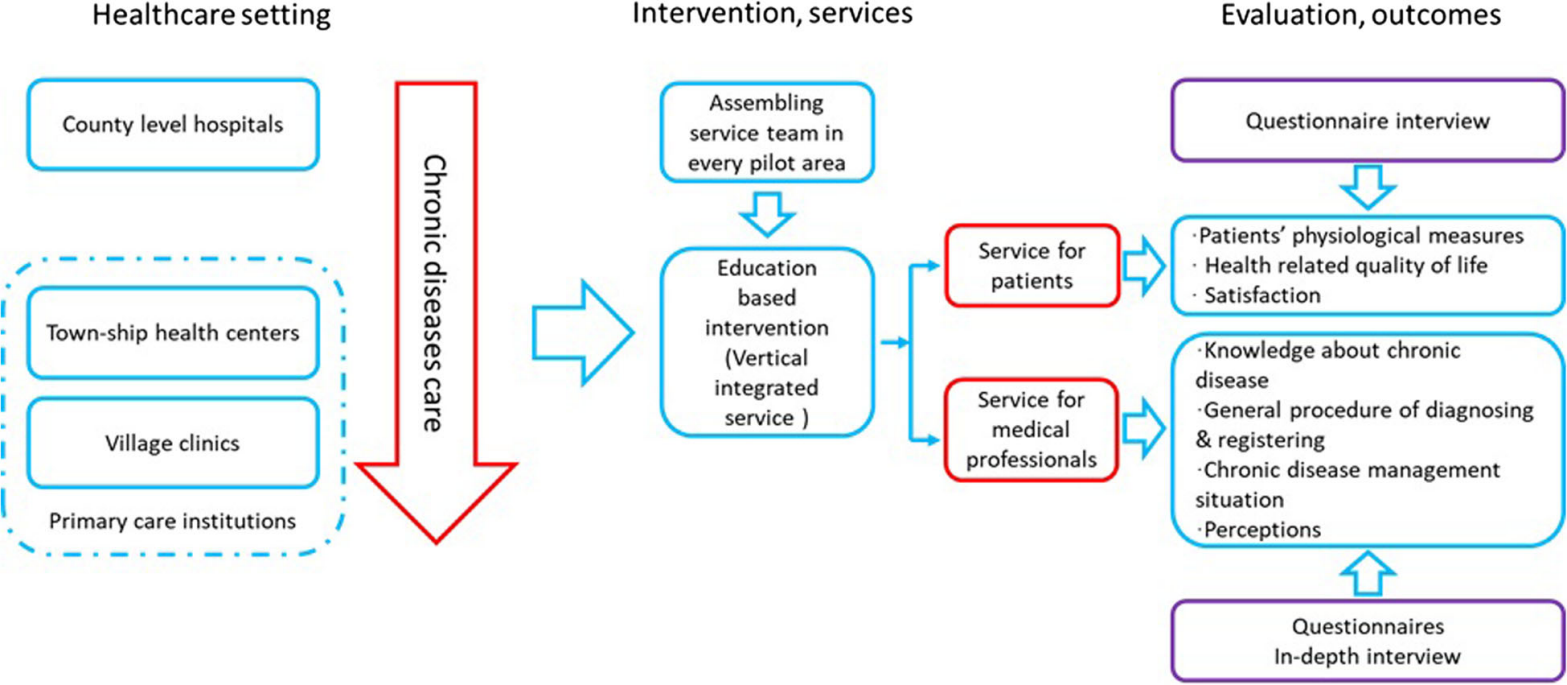
Logic model of the intervention

Figure 1 illustrates the rationale and programme logic of the whole project, including the intervention. As described above, both the financial and mental burden for patients is increasing the need for improving chronic disease management strategies. Therefore, this project has been launched within the context of the ongoing New Round of Medical Reform in China, which aims to optimize the care of patients in rural areas, and to improve the vertical integration in healthcare. An educational based intervention is conducted, based on the model of vertical integrated service between the three-levels of healthcare institutions. Service teams from each pilot county have been assembled to implement the intervention which provides services for both patients and medical professionals. Questionnaire interviews and medical records will be used to collect patients’ data, such as physiological measures, health-related quality of life, and satisfaction with care. Data from medical professionals will be gathered by questionnaires and in-depth interviews covering, for example, knowledge about chronic diseases, the general procedure of diagnosing and registering, chronic disease management situation, and perceptions of chronic disease treatment and prevention. This study protocol has followed the Standard Protocol Items: Recommendations for Interventional Trials (SPIRIT) Guidelines (Additional file 1).

### Study settings (location)

The study was conducted in Jiangsu Province, which is located in the south-east part of China and is one of the high-income provinces. Jiangsu Province can be divided into three parts based on economical and geographical factors: north, middle, and south. The south part is the wealthiest area, whereas the economic status in the middle is worse than the south but better than the north. One county from each part was randomly selected Huaiyin (north), Jingjiang (middle), and Gaochun (south) as pilot areas of the intervention.

After the selection of counties, the local health authorities in the three counties were responsible for selecting the intervention and control townships. In the control townships, the current policy and chronic management were kept. Patients and healthcare professionals who live in the control townships were selected to be comparators. In the three selected counties, 22 townships were included (11 in the intervention group and 11 in the control group). The study population consisted of a convenience sample where all individuals with diabetes and/or hypertension registered at the different village clinics were contacted and asked to visit the clinic at specific dates. All individuals that visited and who met the inclusion criteria were included in the study. Hence, no power calculation has been conducted for this study. We provided towels for patients as gifts to encourage them to participate in this study. Meanwhile, healthcare professionals who volunteer to join in this study received a bonus as a motivation.

### Inclusion and exclusion criteria

The study population consisted of patients with T2DM and primary hypertension from the intervention and control townships in the three pilot counties. The township health centres were responsible for selecting the study population from 1 January 2015 to 1 February 2015. The inclusion and exclusion criteria can be found in Table 1.

**Table 1:** Inclusion and exclusion criteria

Inclusion criteria	
•	Diagnosed with primary hypertension /type 2 diabetes and has been on medications for more than 1 month.^a^
•	Aged 35–75.
•	Has lived in the intervention/control areas for more than 2 years, no records of moving within the last year.
•	Has his/her records on the chronic disease management information system of township health centre or village clinic.
•	Has accepted the chronic disease service provided by township health center or village clinic.
•	Is willing to participate in the project and has a preferable compliance, cognition and receptivity.
Exclusion criteria	
•	Has serious complications related to diabetes or hypertension.
•	Has been diagnosed with secondary hypertension.
•	Has been diagnosed with any other serious disease.
•	Is pregnant or has psychiatric problems.

^a^*.* Hypertension: Blood pressure ≥ 140/90 mmHg; Diabetes: Fasting Blood Glucose (FBG) ≥7.0 mmol/l, and/or 2 h Postprandial Blood Glucose (2hPBG) ≥ 11.1 mmol/l

The study also includes healthcare professionals from different levels of healthcare institutions in the pilot areas. Healthcare professionals from county hospitals, township health centres, and village clinics participated voluntarily. They completed two questionnaires (one for perception of the intervention, and one about assessment of knowledge). For the in-depth interview part, the research team sent invitations to healthcare professionals (including: public health directors, village doctors, directors of hospitals, heads of the health bureaus, heads of health insurance department, and doctors in county hospitals), who can decide whether to take part in the study. Furthermore, the local healthcare institutions including county and township level health bureaus, county level hospitals, township health centres, and village clinics were asked to complete a questionnaire for each healthcare institution.

In total, 4259 patients (2332 with primary hypertension, and 1927 with type 2 diabetes mellitus) agreed to participate in the study. 2114 patients were in the intervention group, and 2145 patients were in the control group. 262 doctors responded to the questionnaires for medical personnel and 235 doctors responded the questionnaires about professional qualification. Eight public health directors, 12 village doctors, seven directors of hospitals, one head of the health bureau, one head of health insurance department and four doctors in county hospitals participated in the in-depth interview. Tables 2 and 3 show the distribution of patient and medical professional participants in the pilot counties.

**Table 2:** Distribution of patient participants by pilot counties and townships

	Intervention group			Control group		
County	Township	Disease		Township	Disease	
		Diabetes (n)	Hypertension (n)		Diabetes (n)	Hypertension (n)
Huaiyin^a^ *(North)*	Nanchenji	185	–	Zhaoji	174	–
	Yangzhuang	173	–	Yuanji	169	–
	Xindu	–	171	Wangxing	–	204
	Lingqiao	–	180	Sanshu	–	191
	Sum	358	351	Sum	343	395
Jingjiang *(Middle)*	Dongxing	97	122	Chengnan	110	130
	Xieqiao	87	151	Huifeng	87	122
	Houhe	83	139	Gushan	88	132
	Sum	267	412	Sum	285	384
Gaochun *(South)*	Qiqiao	86	98	Chunxi	65	132
	Zhuanqiang	87	91	Gubo	80	104
	Yangjiang	90	96	Gucheng	90	85
	Dongba	84	95	Yaxi	92	90
	Sum	347	380		327	411

^a^Due to a different recruitment strategy

**Table 3:** The distribution of healthcare professional participants in pilot counties

Activity	Specific activity	Institution/Professional position	County name		
			Huaiyin	Jingjiang	Gaochun
Questionnaire	Questionnaire for medical personnel	County level hospitals	6	10	5
		Township health centres	56	41	45
		Village clinics	47	25	27
		Sum	109	76	77
	Questionnaire about professional qualification	County level hospitals & township health centres	53	34	44
		Village clinics	50	25	29
		Sum	103	59	73
In-depth interview		Head of the health bureaus	0	0	1
		Public health directors	1	5	3
		Directors of hospitals	1	3	3
		Village doctors	4	7	1
		Doctors in county level hospital	0	2	0
		Doctors in township health centre	0	2	0
		Head of health insurance department	0	0	1
		Sum	6	19	9

### Intervention

The design of this intervention study was to conduct an educational intervention in the pilot counties, and to assess its effects. First, baseline data was collected after which the intervention was conducted by the service teams starting from November 2015. Collection of follow-up data in the pilot counties was done by the research team from October to November 2016. The second follow-up data collection was completed from July to August 2017.

The service team consists of: one consultant, one team manager, one nurse, one public health physician, and one village doctor. A doctor from the county hospital that specializes in treating diabetes or hypertension is the consultant to the service team. One township health centre doctor serves as the team leader. The consultant is responsible for providing technical guidance and training staff and should not serve more than two service teams at the same time.

### Service for patients

Patients in the intervention group received the following services:

- Lectures every 2 months mainly focusing on prevention and self-management strategies for chronic disease, nutrition and physical activity, proper health behaviours, and psychological counselling.
- Periodical follow-up interviews every 2 months along with an annual physical examination.
- Special medical service, including helping patients with medical treatment, transfer treatment, return visit, and clinical care, etc.

Participants in the control group received routine services as usual.

### Service for medical professionals

Healthcare professionals in the intervention group received the following services:

- Training lessons for village GPs every 2 months.
- Regular meetings every 2 months to discuss their work progress.
- A weekly team communication about analysing patients’ conditions or formulating personalized therapeutic regimens.
- Technical checks to inspect the patients’ disease monitoring schemes, prevention and treatment plans in every 2 months.
- New performance appraisals for doctors and nurses were implemented in intervention townships, containing two parts: 1) Bonus for those who do well in the project, 2) Opportunities for professional development for outstanding doctors or nurses.

For healthcare professionals in the control areas, no service team was assembled and current routine health services continued as usual.

### Quality control

The local authority (county level health bureaus and county level Centres for Disease Control) will take charge of quality control process. Three main measures will be taken by those institutions.

- All service teams should participate in a compulsory training course before the intervention. The training course contains guidance and instructions about implementing the intervention, such as assembling the service team, the duty of the members and different institutions, the service objectives, and the intervention details. The training course aims to ensure that all participating patients will receive the same service from different service teams.
- Information about all participating patients will be recorded in the same format by service teams. The name of the participant, their disease, and their contact number will be collected, in order to check if there is any loss to follow-up.
- The last part is the consistency criteria of performance appraisal by the local authorities for all the medical professionals in the intervention townships. The project encourages the staff to actively cooperate with the researchers and this criterion will offer rewards (bonus and chance of promotion) for those who perform excellently during the intervention.

### Data collection

#### Primary outcome (patients)

1. Patients’ physiological measures: blood glucose/glycosylated haemoglobin (HbAlc)/blood pressure.
2. Health status measured by the generic health-related quality of life instrument EQ-5D [35].
3. Satisfaction with health services, health insurance, understanding of diabetes, health seeking behaviour and patients’ out-of-pocket costs of care.

#### Secondary outcomes (healthcare professionals)

1. Knowledge about the current epidemiological conditions of chronic disease in their own village.
2. General procedure of diagnosing and registering chronic disease.
3. Quality of chronic disease management before and after intervention.
4. Healthcare professionals’ general perceptions about the intervention.

### Data monitoring

A Data Monitoring Committee (DMC) has not been established in this study, due to the fact that no unusual high safety concerns have been raised up related to the participants. The intervention in this study is an educational intervention, which means that is does not affect the medical treatment. No interim analysis has been conducted, and we have no plan of collecting information about adverse events or other unintended effects. The recruitment of patients will be carefully supervised by the Ethics Committee of Nanjing Medical University.

### Data analysis

The structured interview with healthcare professionals will be tape recorded and transcribed. Transcriptions will be analysed using conventional content analysis [36]. Patient questionnaire data will be analysed for differences between patient groups in intervention and control counties, before and after the intervention, by using Mann-Whitney U-test. A longitudinal model will be used to test the changes in blood glucose/blood pressure control rate and HbA1c control rate during the follow-up. For EQ-5D, independent samples t-test will be performed to study differences in mean EQ-5Dindex and EQ VAS score. Multivariate regression will be performed to identify factors predicting variation in mean EQ-5D index and EQ VAS score.

## Preliminary results

Analysis of baseline data on socioeconomic variables of all patient participants was conducted in order to assess differences in those variables among patients in the intervention and control groups (Table 4). At the 95% confidence level, there was no difference in the included socioeconomic variables (sex, marital status, education level, occupation, healthcare insurance type) between intervention and control groups for patients with T2DM and hypertension.

**Table 4:** Baseline data analysis on the socioeconomic variables for patients

		Hypertension					Diabetes				
		Intervention		Control		*P*-value	Intervention		Control		*P*-value
		N	%	N	%		N	%	N	%	
Mean (SD) age		62.21 (8.17)		62.70 (8.14)		0.24	61.95 (8.36)		61.35 (8.90)		0.23
Sex	Male	329	43.5	309	40.4	0.23	193	30.2	180	31.3	0.71
	female	427	56.5	455	59.6		446	69.8	396	68.8	
Marital status	Single	104	13.8	126	16.5	0.15	82	12.9	84	14.6	0.40
	Married	649	86.2	637	83.5		566	87.1	490	85.4	
Education	Lower education^a^	518	68.6	550	72.2	0.13	465	72.8	420	73.0	0.95
	Higher education^b^	237	31.4	212	27.8		174	27.2	155	27.0	
Occupation	Farming or house working	616	81.5	645	84.8	0.10	520	81.6	481	83.8	0.32
	Non-farming or others	140	18.5	116	15.2		117	18.4	93	16.2	
Healthcare insurance	NRCMS^c^	704	93.1	678	89.2	0.24	571	89.5	493	86.2	0.37
	UEMI^d^	19	2.5	52	6.8		23	3.6	43	7.5	
	URMI^e^	21	2.8	21	2.8		38	6.0	29	5.1	
	Other types of insurance or no insurance	12	1.6	9	1.2		6	0.9	7	1.2	

^a^. *Lower education* Primary school education or lower

^b^. *Higher education* Middle school education or higher

^c^. *NRCMS* New Rural Cooperative Medical System

^d^. *UEMI* Urban Employee Medical Insurance

^e^. *URMI* Urban Residents Medical Insurance

## Discussion

Considering the current strategies for chronic disease, the ultimate aim of the intervention is to establish an effective management mechanism for chronic patients in rural China. The intervention is based on the theory of vertical integrated service model in healthcare [24, 26], but there is a lack of experience of conducting such interventions in rural China [37]. Hence, it is necessary to conduct such a study for chronic patients who live in the rural areas, especially as the central government has put chronic disease management into the New Round of Medical Reform blueprints [15]. Meanwhile, another plan named Healthy China 2020 has been reviewed and approved in August 2016 by the Political Bureau in the Communist Party of China Central Committee, which emphasized the necessity and urgency of building and reforming a modern management mechanism for different types of diseases [38]. This study will also contribute to this national plan.

Currently, the study is at the earlier stages of data analysis. The intervention has been conducted smoothly and been positively accepted by local participants so far. Patients with hypertension and diabetes can benefit from the intervention by receiving better follow-up services and useful information about their diseases. They are, therefore, willing to participate and actively respond to the questionnaire. For healthcare professionals in the pilot counties, the intervention has raised a lot of attention in all three levels of healthcare institutions, and most of the healthcare professionals are glad to see the reforming and strengthening of the current chronic disease management strategies. Meanwhile, the intervention has been strongly supported by local health authorities, who are already on their way of exploring the appropriate way of providing high quality and efficient healthcare service for all patients.

Methodological challenges still remain, especially in the process of data collecting and analysing. For example, as many participants have low education, the questions must be phrased so that they are easy to understand. However, great care is taken to make sure the meaning of translation is exactly the same as the original question. A supervision team shall be established to strictly assess all the research activities from the perspective of ethics.

The research team anticipate positive results: the patients’ physical health outcome as well as health-related quality of life can be improved after the intervention. Furthermore, their satisfaction with health services, understanding of diabetes, health seeking behaviour will improve, and patients’ out-of-pocket costs of care will be reduced. The healthcare professionals who participate in the project can acquire more knowledge about the prevention and treatment for chronic disease. Furthermore, we hope that the positive changes mentioned above can be shown to be associated with the intervention implementation.

## Additional file

Additional file 1: SPIRIT figure. Study design. (DOC 124 kb)

## Abbreviations

CCM: Collaborative care model
NHFPC: National Health and Family Planning Commission
NNSFC: National Natural Science Foundation of China
NRCMS: New Rural Cooperative Medical Service
T2DM: Type 2 diabetes mellitus
VA: Veterans affairs
