# Supplementary material for: Vertical integrated service model: an educational intervention for chronic disease management and its effects in rural China – a study protocol
Source: BMC Health Serv Res. 2018 Jul 20;18:567. doi: 10.1186/s12913-018-3355-8 (PMC6053730; doi:10.1186/s12913-018-3355-8)
Supplement: Supplementary file 1 — SPIRIT figure. Study design. (DOC 124 kb) [file 12913_2018_3355_MOESM1_ESM.doc]

Figure. The schedule of enrolment, interventions, and assessments

|  | **STUDY PERIOD** | | | | |
| --- | --- | --- | --- | --- | --- |
|  | **Enrolment** | **Allocation** | **Post-allocation** | | |
| **TIMEPOINT**** | ***Oct. 2015*** | ***Oct. 2015*** | ***Nov. 2015*** | ***Nov. 2016*** | ***Aug. 2017*** |
| **ENROLMENT:** |  |  |  |  |  |
| **Eligibility screen** | X |  |  |  |  |
| **Informed consent** | X |  |  |  |  |
| **Allocation** |  | X |  |  |  |
| **INTERVENTIONS:** |  |  |  |  |  |
| ***Service for patients*** |  |  |  |  |  |
| ***Service for healthcare professionals*** |  |  |  |  |  |
| **ASSESSMENTS:** |  |  |  |  |  |
| ***Patients’ socio-economic data*** |  |  | X |  |  |
| ***Primary outcome (patients)*** |  |  | X | X | X |
| ***Secondary outcomes (healthcare professionals)*** |  |  | X | X | X |
